# Alterations in Intestinal Brush Border Membrane Functionality and Bacterial Populations Following Intra-Amniotic Administration (*Gallus gallus*) of Catechin and Its Derivatives

**DOI:** 10.3390/nu14193924

**Published:** 2022-09-22

**Authors:** Nikolai Kolba, Amin Zarei, Jacquelyn Cheng, Nikita Agarwal, Younas Dadmohammadi, Leila Khazdooz, Alireza Abbaspourrad, Elad Tako

**Affiliations:** Department of Food Science, Cornell University, Ithaca, NY 14853, USA

**Keywords:** intra-amniotic administration, brush border membrane, catechin derivatives, microbiome

## Abstract

Catechin is a flavonoid naturally present in numerous dietary products and fruits (e.g., apples, berries, grape seeds, kiwis, green tea, red wine, etc.) and has previously been shown to be an antioxidant and beneficial for the gut microbiome. To further enhance the health benefits, bioavailability, and stability of catechin, we synthesized and characterized catechin pentaacetate and catechin pentabutanoate as two new ester derivatives of catechin. Catechin and its derivatives were assessed in vivo via intra-amniotic administration (*Gallus gallus*), with the following treatment groups: (1) non-injected (control); (2) deionized H_2_O (control); (3) Tween (0.004 mg/mL dose); (4) inulin (50 mg/mL dose); (5) Catechin (6.2 mg/mL dose); (6) Catechin pentaacetate (10 mg/mL dose); and (7) Catechin pentabutanoate (12.8 mg/mL dose). The effects on physiological markers associated with brush border membrane morphology, intestinal bacterial populations, and duodenal gene expression of key proteins were investigated. Compared to the controls, our results demonstrated a significant (*p* < 0.05) decrease in *Clostridium* genera and *E. coli* species density with catechin and its synthetic derivative exposure. Furthermore, catechin and its derivatives decreased iron and zinc transporter (Ferroportin and ZnT1, respectively) gene expression in the duodenum compared to the controls. In conclusion, catechin and its synthetic derivatives have the potential to improve intestinal morphology and functionality and positively modulate the microbiome.

## 1. Introduction

Catechins are potent polyphenolic compounds naturally present in plant-based foods such as vegetables, beverages, green tea, and fruits such as cherries, grapes, apples, and pears [1]. Like other flavonoids, catechins possess a multitude of biological and pharmaceutical properties that can improve human health and prevent various diseases. For example, they can inhibit the activities of various reactive oxygen species (ROS) by preventing damage to proteins, DNA, and cell membranes [2,3]. Compared to antioxidants such as ascorbic acid, α-tocopherol, curcumin, and butylated hydroxyanisole, catechins are more effective at scavenging free radicals [1,4]. The antioxidant capacity of catechins has previously been shown to be powerful inhibitors against various cancers and tumors in humans and animals [4,5,6,7]. Due to high antioxidant activity, these compounds have been reported to inhibit the oxidation of low-density lipoproteins (LDL), preventing plaque formation and decreasing cardiovascular disease incidence [8,9,10]. Catechins have also been shown to possess other beneficial and protective properties, such as anti-inflammatory, anti-HIV, anti-aging, anti-allergic, anti-diabetic, and anti-bacterial activities [11,12,13,14,15,16,17,18]. Moreover, these compounds exhibit noteworthy neuroprotection against neurodegenerative diseases such as Alzheimer’s disease, Parkinson’s disease, and ischemic damage [19,20].

Recent studies have demonstrated that polyphenolic compounds can be used as prebiotics to improve the structure and function of the gut microbiota [21,22]. Dietary polyphenolic compounds can be transformed via intestinal microbiota fermentation into short-chain fatty acids (SCFAs) [23,24,25,26]. SCFAs are a source of energy for colonic cells, where increased SCFA production has been associated with increased populations of beneficial bacteria, acidification of the intestinal lumen, and, consequently, the inhibition of harmful pathogen growth [27]. Acetate and butyrate are two key SCFAs utilized as a source of energy for epithelial cells to improve intestinal barrier integrity and prevent the translocation of antigens and pathogens into circulation [28]. Moreover, increased SCFA production has been associated with increases in villus surface area, positive modulations in gut microbiota composition, and enhanced functionality of the duodenal brush border membrane (BBM) [29].

While catechin consumption has been found to possess a myriad of beneficial physiological effects, low stability (i.e., pH, thermal, storage) and permeability of catechins in body membranes limit their bioavailability [1]. Further, catechins can be significantly degraded in the key site of human digestion, the small intestine, where ROS, digestive secretions, and changes in pH exacerbate catechin autoxidation and degradation [30]. Thus, microencapsulation and chemical modification can improve catechin stability and bioavailability during digestion [1,31,32]. In this vein, a purposeful chemical modification of natural polyphenolic compounds, such as adding short-chain ester groups, can increase compound stability. Moreover, probiotic bacterial populations can hydrolyze and metabolize these short-chain ester groups to produce beneficial SCFAs.

**Scheme 1:**
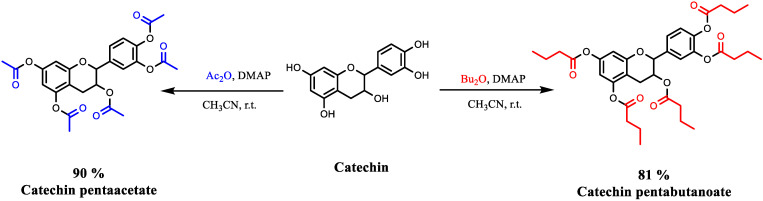
Synthesis of catechin pentaacetate and catechin pentabutanoate. The reported percentages are the reaction yield.

In this work, we reported the modification of catechin by converting it to catechin pentabutanoate (a new molecule that has not been previously reported) and catechin pentaacetate as two types of catechin short-chain ester derivatives (Scheme 1). Further, to characterize the physiological effects of catechin and its short-chain ester derivatives on brush border membrane (BBM) functionality, intestinal morphology, and intestinal microbial populations, we utilized the in vivo *Gallus gallus* model. The *Gallus gallus* has been used as a novel and cost-effective animal model to elucidate the physiological effects of plant bioactives and nutritional solutions relevant to human nutrition [33,34,35,36,37,38,39,40]. Recently, the positive impact of catechins, as food additives, on poultry growth performance and egg quality has been studied and found to improve poultry antioxidant status [41,42]. Our present study utilizes the intra-amniotic administration approach (*in ovo* feeding) in the *Gallus gallus*, where the amniotic fluid, which is naturally and orally consumed by the embryo starting at day 17, is entirely consumed by hatch, which allows for testing the effects of the solution administered into the amniotic fluid on the different systems of interest [29,39,43,44,45]. In the current study, the impact of intra-amniotic administration of catechin and its derivatives at a dosage range of 6.2–12.8 mg/mL on BBM functionality was assessed by evaluating duodenal gene expression of biomarkers of mineral status, BBM digestive and absorptive ability, immune function, and inflammation in vivo in the *Gallus gallus*. A secondary objective was to evaluate the effects of the intra-amniotic administration of catechin and its derivatives on cecal bacterial populations by quantifying the relative abundances of health-promoting populations (*Bifidobacterium* spp. and *Lactobacillus* spp.) versus those of potentially pathogenic bacteria (*E. coli* and *Clostridium* spp.). We hypothesize that catechin and its more stable derivatives, when administered intra-amniotically, will cause favorable alterations in BBM functionality and development and positively modulate the gut microbial populations, where increased effects will be seen with catechin derivatives due to increased stability within the gastrointestinal tract as was demonstrated in our previous work [46].

## 2. Materials and Methods

### 2.1. Animals

Cornish cross-fertile broiler chicken eggs (*n* = 41) were obtained from a commercial hatchery (Moyer’s chicks, Quakertown, PA, USA). The eggs were incubated at the Cornell University Animal Science poultry farm incubator under optimal conditions. All animal protocols were approved by Cornell University Institutional Animal Care and Use Committee (IACUC #2020-0077).

### 2.2. Materials

(+)-Catechin and butyric anhydride with 98% purity and 4-dimethylamino pyridine with 99% purity were purchased from Sigma Aldrich (St. Louis, MO, USA). Acetic anhydride with 99% purity was purchased from Acros (Morris Plains, NJ, USA). Silica gel (P60, 40–63 µm, 60 Å) was purchased from SiliCycle (Quebec, QC, Canada), and Silica Gel 60 F254 Coated Aluminum-Backed TLC (thin layer chromatography) sheets were purchased from EMD Millipore (Billerica, MA, USA).

### 2.3. General Procedure for the Synthesis of Catechin Pentabutanoate

To a round bottom flask with a magnetic stir bar, septa, and nitrogen inlet, 200 mg (0.65 mmol) of catechin.H_2_O, 2.1 mL (12.6 mmol) of butyric anhydride, 35 mg (0.28 mmol) of 4-dimethylamino pyridine, and 5 mL of acetonitrile were added. The reaction mixture was stirred at room temperature for 24 h under a nitrogen atmosphere. The progress of the reaction was followed by thin-layer chromatography (TLC). After the reaction, the excess amount of butyric anhydride was evaporated, and the crude product was extracted with 30 mL of ethyl acetate. Then, the organic phase was washed with (4 × 20 mL) of HCl (2 M). After that, the organic phase was washed with (3 × 20 mL) of NaHCO_3_ (0.4 M). Finally, the organic solvent was dried with sodium sulfate and evaporated by a rotary evaporator to obtain the product in 81% yield (337 mg) as a light-yellow viscose liquid (λ_max_ in ethanol was 271 nm).

### 2.4. General Procedure for the Synthesis of Catechin Pentaacetate

To a round bottom flask with a magnetic stir bar, septa, and nitrogen inlet, 200 mg (0.65 mmol) of catechin.H_2_O, 1.2 mL (12.6 mmol) of acetic anhydride, 35 mg (0.28 mmol) of 4-dimethylamino pyridine, and 5 mL of acetonitrile were added. The reaction mixture was stirred at room temperature for 24 h under a nitrogen atmosphere. TLC followed the progress of the reaction. After the reaction, the excess acetic anhydride was evaporated, and the crude product was extracted with 30 mL of ethyl acetate. Then, the organic phase was washed with (4 × 20 mL) of HCl (2 M). After that, the organic phase was washed with (3 × 20 mL) of NaHCO_3_ (0.4 M). Finally, the organic solvent was dried with sodium sulfate and evaporated by a rotary evaporator to obtain the product in 90% yield (293 mg) as a light-yellow viscose liquid (λ_max_ in ethanol was 270 nm).

### 2.5. Characterization of Catechin Pentabutanoate and Catechin Pentaacetate

#### 2.5.1. ^1^H NMR (500 MHz) and ^13^C NMR

A 500 MHz NMR (Bruker AVANCE) spectrometer was used for ^1^H NMR (500 MHz) and ^13^C NMR (125 MHz) spectra in CDCl_3_. The chemical shifts were expressed in δ (ppm) relative to Tetramethylsilane (TMS) as the internal standard, and coupling constants (*J*) were measured in Hz. Spin multiplicities were described as singlet (s), doublet (d), triplet (t), quartet (q), sextet (sext), and multiplet (m).

#### 2.5.2. Fourier Transform Infrared Spectroscopy

Fourier transform infrared spectra (ATR-FTIR) were recorded on a Shimadzu IRAffinity-1S spectrophotometer.

#### 2.5.3. Ultraviolet–Visible Spectroscopy (UV-Vis)

UV-vis was recorded on a Shimadzu UV-2600 spectrophotometer, as was previously described [32,46].

#### 2.5.4. Liquid Chromatography-Mass Spectrometry Analysis

For LC-MS analysis, we used an LC (Agilent 1100 series) coupled with a mass spectrometer. All samples were passed through a 13 mm nylon syringe filter with a 0.22 μm pore size before injection to ensure the removal of the solid contaminants. Reverse-phase chromatography was used with a Phenomenex Luna Omega (Phenomenex) LC column with the following specifications: 100 × 4.6 mm, 3 µm, polar C18, 100 Å pore size with a flow rate of 0.3 mL min^−1^. LC eluents include MiliQ-H_2_O containing 0.1 % formic acid (15%) and acetonitrile (85%) using isocratic elution. The time of analysis was 20 min. The mass spectrometer (Finnigan LTQ mass spectrometer) was equipped with an electrospray interface (ESI) set in positive electrospray ionization mode for analysis and with 15 kV collision energy.

#### 2.5.5. Particle Size Measurement

To ensure nanosize particles for intra-amniotic administration, the particle size distribution and mean particle diameter (Zeta average size) of catechin pentabutanoate and catechin pentaacetate in DI H_2_O containing 0.4% (*w*/*v*) tween 80 were measured using a commercial dynamic light-scattering device (Nano-ZS, Malvern Instruments, Worcestershire, UK).

### 2.6. Preparation of Catechin Solution (0.02 M)

0.31 g of catechin was dissolved in 50 mL of DI H_2_O containing 2% (*v*/*v*) EtOH to prepare a 0.02 mol/L catechin solution.

### 2.7. Dispersion of Catechin Pentabutanoate and Catechin Pentaacetate in DI H_2_O

One mmol of catechin pentabutanoate or catechin pentaacetate was dissolved in 2 mL of pure EtOH, and then this phase was added to the 50 mL of DI H_2_O containing 0.4% (*w*/*v*) tween 80 and homogenized at room temperature at 15,000 rpm. The total concentration of catechin pentaacetate in this emulsion was approximately 0.02 mol/L.

### 2.8. Intra-Amniotic Administration

The intra-amniotic administration procedure was previously described by Tako et al. [47]. On Day 17 of embryonic incubation, eggs with viable embryos were weighed and divided into seven groups (*n* = 10) with approximately equal weight distribution. The seven groups were assigned: (1) non-injected (control); (2) deionized H_2_O (control); (3) Tween (0.004 mg/mL dose); (4) inulin (50 mg/mL dose); (5) catechin (6.2 mg/mL dose); (6) catechin pentaacetate (10 mg/mL dose); and (7) catechin pentabutanoate (12.8 mg/mL dose). The intra-amniotic injection solution (1 mL per egg) was injected with a 21-gauge needle into the amniotic fluid, identified by candling. Following the injection, the injection sites were sealed with cellophane tape. Eggs were then placed in hatching baskets, with each treatment equally represented at each incubator location.

### 2.9. Blood and Tissue Collection

As previously described [48], immediately following hatching (Day 21), birds were weighed and euthanized with CO_2_ exposure. Blood was collected using micro-hematocrit heparinized capillary tubes (Thermo Fisher Scientific, Waltham, MA, USA). The small intestines, ceca, and livers were quickly removed from the carcasses and placed in separate sterile cryovials (Simport, Beloeil, QC, Canada) for storage. Ceca were weighed before storage. The samples were immediately frozen in liquid nitrogen and stored at −80 °C until analysis [49].

### 2.10. Blood Hemoglobin Measurements

Blood Hemoglobin (Hb) concentrations were determined spectrophotometrically using the QuantiChromTM Hemoglobin Assay (DIHB-250, BioAssay Systems, Hayward, CA, USA) following the kit manufacturer’s instructions.

### 2.11. Isolation of Total RNA From Chicken Duodenum

Total RNA was extracted from 30 mg of the proximal duodenal tissue using a Qiagen RNeasy Mini Kit (Qiagen Inc., Germantown, MD) according to the manufacturer’s protocol. Total RNA was eluted in 50 μL of RNase-free H_2_O. All steps were carried out under RNase-free conditions. RNA was quantified with a NanoDrop 2000 (ThermoFisher Scientific, Waltham, MA, USA) at *A_260/280_*. RNA was stored at −80 °C until use.

### 2.12. Real-Time Polymerase Chain Reaction

As previously described [34,43,44,46], the primers used in the real-time polymerase chain reactions (RT-PCR) were designed using Real-Time Primer Design Tool software (IDT DNA, Coralvilla, IA, USA) based on 13 gene sequences from the GenBank database. The sequences are shown in Table S1. The amplicon length was limited to 90 to 150 bp, the size of the primers was from 17-to 25-mer, and the GC content was between 41 and 55%. The specificity of the primers was tested by performing a BLAST search against the genomic NCBI database.

cDNA was generated using a C1000 Touch thermocycler (Biorad, Hercules, CA, USA) and a Promega-Improm-II Reverse Transcriptase Kit (Catalog #A1250) 20 μL reverse transcriptase reaction. The reverse transcriptase reaction consisted of 1 μg total RNA template, ten μM random hexamer primers, and two mM of oligo-dT primers. All reactions were performed under the following conditions: 94 °C for 5 min, 60 min at 42 °C, 70 °C for 15 min, and hold at 4 °C. The concentration of cDNA obtained was determined with a NanoDrop 2000 at *A_260/280_* with an extinction coefficient of 33 for single-stranded DNA.

RT-PCR was performed with a Bio-RadCFX96 Touch (Hercules, CA, USA). The ten μL RT-PCR mixtures consisted of cDNA (2 μg), 2X BioRad SSO Advanced Universal SYBR Green Supermix (Cat #1725274, Hercules, CA, USA), forward and reversed primers, and nuclease-free H_2_O (for the no template control). The no-template control of nuclease-free H_2_O was included to exclude DNA contamination in the PCR mix. All reactions were performed in duplicates and under the following conditions: initial denaturing at 95 °C for 30 s, 40 cycles of denaturing at 95 °C for 15 s, various annealing temperatures according to IDT for 30 s and elongating at 60 °C for 30 s. After the cycling process was completed, melting curves were determined from 65.0 °C to 95.0 °C with increments of 0.5 °C for 5 s to ensure the amplification of a single product. RT-PCR efficiency values for the eleven genes were as follows: DcytB, 1.046; DMT1, 0.998; Ferroportin, 1.109; ZIP9, 1.035; ZnT1, 1.09; ZnT7, 1.013; SGLT-1, 0.994; SI, 1.032; AP, 1.015; Muc2, 1.102; NF-κβ1, 1.113; TNF-α, 1.046; IL8, 0.998; 18s rRNA, 0.994. Gene expression levels were obtained from Ct values based on the ‘second derivative maximum’ computed by the Bio-Rad CFX Maestro Software Version 2.2 (Bio-Rad, Hercules, CA, USA). Gene expression was normalized to the expression of 18S rRNA.

### 2.13. Collection of Microbial Samples and Intestinal Contents DNA Isolation

The contents of the ceca were placed into a sterile 15 mL tube (Corning, Corning, NY, USA) containing 9 mL of sterile 1X phosphate-buffered saline (PBS) and homogenized by vortexing with glass beads (3 mm diameter) for 3 min. Debris was removed by centrifugation at 700× *g* for 1 min, and the supernatant was collected and centrifuged at 12,000× *g* for 5 min. The pellet was washed twice with 1X PBS and stored at −20 °C until DNA extraction.

The pellet was re-suspended in 50 mM EDTA to extract DNA and treated with 10 mg/mL lysozyme (Sigma Aldrich CO., St. Louis, MO, USA) for 45 min at 37 °C. The bacterial genomic DNA was then isolated using a Wizard Genomic DNA purification kit (Promega Corp., Madison, WI, USA), following the manufacturer’s instructions.

### 2.14. PCR Amplification of Bacterial 16s rDNA

Primers for *Lactobacillus*, *Bifidobacterium*, *Clostridium*, and *E. coli* were designed according to previously published data by Zhu et al. [50]. The universal primers, which identify all known strains of bacteria in the intestine, were prepared with the invariant region in the 16S rRNA of bacteria and used as an internal standard to normalize the results. PCR products were separated by electrophoresis on 2% agarose gel, stained with ethidium bromide, and quantified using the Quantity One 1-D analysis software (BioRad, Hercules, CA, USA).

### 2.15. Glycogen Analysis

Glycogen analysis was obtained from the pectoralis muscle, and liver previously described [51]. Briefly, the frozen pectoral muscle or liver samples were homogenized in 8% perchloric acid. Pectoral muscle samples were centrifuged at 12,000× *g* for 15 min, and liver samples were centrifuged at 4000× *g* for 15 min at 4 °C. The supernatant was removed, and 1.0 mL of petroleum ether was added to each tube. After mixing, the petroleum ether fraction was removed, and samples from the bottom layer were transferred to a 96-well plate containing 300 μL of an iodine color reagent. All samples were read at a wavelength of 450 nm in a microplate spectrophotometer (Epoch, BioTek, VT, USA), and the amount of glycogen was calculated according to a standard curve. The amount of glycogen present in the pectoral sample was determined by multiplying the weight of the tissue by the amount of glycogen per 1 g of wet tissue.

### 2.16. Morphometric Examination of Duodenal Tissue

Villus epithelium analysis was conducted as previously published [43,44,52,53]. The duodenal samples were soaked in buffered formaldehyde (4% (*v*/*v*)), dehydrated, cleared, and embedded in paraffin. Numerous sections were cut with a thickness of 5 µm and put on slides. The sections were deparaffinized in xylene, after which they were rehydrated in a series of graded alcohol. Finally, the slides were stained with Alcian Blue–periodic acid-Schiff and investigated under light microscopy. The variables were assessed (light microscope, EPIX XCAP software, standard version, Olympus, Waltham, MA, USA) for the following: villus length, villus diameter, depth of crypts, goblet cell diameter, crypt goblet cell number, and villus goblet cells type number (acidic, neutral, or mixed). Four segments were examined for each biological sample and five biological samples per treatment group. The goblet cells were enumerated at ten villi/samples, and the means were calculated for statistical analysis.

### 2.17. Statistical Analysis

Experimental treatments for the intraamniotic administration assay were arranged in a completely randomized design. Initially, data was investigated for normality utilizing the Kolmogorov–Smirnov test. Results were analyzed by one-way multiple analysis of variance (ANOVA). Statistical analyses were carried out using SPSS version 27.0 software. Differences between treatment groups were compared with a post hoc Duncan test, with statistically different results at *p* < 0.05. Results are expressed as mean ± standard error, *n* ≥ 8.

## 3. Results

After chemical modification of catechin-to-catechin pentabutanoate and catechin pentaacetate, the structures of these compounds were characterized by FTIR, ^1^H NMR, ^13^C NMR, and LC-MS in the following orders:

### 3.1. Fourier-Transform Infrared (FTIR) Spectroscopy of Catechin Pentabutanoate

In the structure of catechin pentabutanoate, there are four butanoate chains bonded to aromatic rings. A strong band at 1761 cm^−1^ confirms the carbonyl of these ester groups. Another band at 1739 cm^−1^ demonstrates the presence of the aliphatic ester group in this molecule. FTIR results show that two bands at 2966 and 2875 cm^−1^ are attributed to asymmetric and symmetric stretching vibrations of aliphatic C-H, respectively (Figure S1). The stretching vibrations of C=C bonds in aromatic rings appear at 1622 and 1595, 1506, and 1489 cm^−1^. The bands at 1458 and 1361 cm^−1^ are the aliphatic C-H bending vibrations of methylene and methyl groups, respectively. The stretching vibrations of C-O bonds appear at 1242 and 1122 cm^−1^ as strong and broadband. Finally, the bands at 916, 837, 800, and 748 cm^−1^ are evidence of the out-of-plane C-H bending vibrations of the aromatic rings (Figure S1).

### 3.2. ^1^H NMR of Catechin Pentabutanoate

The ^1^H NMR (500 MHz) of catechin pentabutanoate was performed in CDCl_3_ at room temperature (Figure S2). The expanded ^1^H NMR of this compound showed that the most deshielded proton (H5′) at 7.25 ppm that appeared as a doublet of doublets peak (*J*_1_ = 8.5 Hz, *J*_2_ = 2.5 Hz) was attributed to the aromatic hydrogen located on the aromatic ring at carbon 5′ (Figure S3). The chemical shifts of two aromatic hydrogens (H4′ and H1′) were so close together that they overlapped and appeared as a doublet peak at 7.19 ppm. The other aromatic hydrogens (H8 and H6) were observed in this compound as two doublet peaks (*J* = 2 Hz) at 6.66 and 6.59 ppm, respectively. A quartet peak (*J* = 7 Hz) at 5.28 ppm was attributed to H3 located on the aliphatic ring in the structure of catechin pentabutanoate. Another hydrogen on the aliphatic ring is H2 which appeared at 5.14 ppm as a doublet peak (*J* = 6.5 Hz). In this molecule, there were two diastereotopic hydrogens (H4) at 2.90 ppm (*J*_1_ = 16.75 Hz, *J*_2_ = 5.5 Hz) and 2.66 ppm (*J*_1_ = 16.75 Hz, *J*_2_ = 6.5 Hz) as two doublets of doublets patterns (*J*_1_ = 16.5 Hz, *J*_2_ = 6 Hz).

In the five chain ester groups of catechin pentabutanoate, four CH_2_ groups near the ester carbonyl groups bonded to aromatic rings, which appeared as multiplets between 2.55–2.51 ppm with integral 8 (Figure S4). The chemical shift of another CH_2_ group in the vicinity of an ester carbonyl group bonded to an aliphatic ring was 2.23 ppm as a triplet peak (*J* = 7.5 Hz). In those ester chains bonded to the aromatic rings, a multiplet peak at 1.78 ppm was attributed to the four methylene groups near the methyl groups. It should be mentioned that in another ester chain bonded to the aliphatic ring, methylene is in the neighborhood of a methyl group that appeared at 1.55 ppm as a sextet (*J* = 7.5 Hz). A multiplet peak at 1.05 ppm with an integral of 12 confirmed the existence of four methyl groups at the end of the butanoate esters arms bonded to the aromatic rings. Finally, a triplet peak (*J* = 7.5 Hz) at 0.85 ppm was attributed to a CH_3_ group at the end of the butanoate ester chain bonded to the aliphatic ring.

### 3.3. ^13^C NMR of Catechin Pentabutanoate

The ^13^C NMR (125 MHz) of catechin pentabutanoate in CDCl_3_ was also studied at room temperature (Figure S5). The ^13^C NMR of this compound revealed five peaks at 172.76, 171.66, 171.07, 170.74, and 170.72 ppm attributed to the five different carbonyl carbons of the ester groups in the structure of this molecule. There are twelve distinct peaks at 154.46, 149.92, 149.47, 142.24, 142.20, 136.03, 124.41, 123.70, 121.87, 110.18, 108.79, and 107.60 ppm for the carbons of the aromatic rings. In the aliphatic ring of catechin pentabutanoate, two different carbons bonded to ether and ester oxygens (C2 and C3) appear at 77.82 and 68.14 ppm, respectively. Another carbon in the aliphatic ring was a benzylic methylene carbon (C4) with a chemical shift of 24.18 ppm. In the five short ester chains of catechin pentabutanoate, five dissimilar peaks at 36.19, 36.03, 36.00, 35.89, and 35.84 ppm were attributed to the five CH_2_ groups near the carbonyl carbons of the ester groups (Figure S5). The rest of the methylene groups in these chains appeared at 18.44, 18.41, 18.39, 18.37, and 18.25 ppm. In this compound, five methyl groups were at the end of ester chains. Because the chemical shifts of these methyl groups were close, one of the methyl groups overlapped with another, resulting in these five methyl groups appearing as four peaks at 13.67, 13.64, 13.63, and 13.42 ppm (Figures S5 and S6).

### 3.4. LC-MS of Catechin Pentabutanoate

For further assurance of the synthesis and purity of catechin pentabutanoate, LC-MS was performed to determine the molecular weight of catechin pentabutanoate (Figure S7). The results of selected reaction monitoring (SRM) show a single peak with 641.22 m/z (M + H), which agrees with the structure and molecular weight of catechin pentabutanoate. A fragment with 553.04 m/z is attributed to eliminating a butanoic acid molecule from the catechin pentabutanoate.

### 3.5. Particle Sizes of Catechin Pentabutanoate

Catechin pentabutanoate was dispersed in DI H_2_O using 2% (*v*/*v*) ethanol as a cosolvent and 0.4 % (*w*/*v*) of tween 80 as a surfactant. The average size of the catechin pentabutanoate particles was 232 nm, and the polydispersity index (PDI) was 0.185.

### 3.6. FTIR of Catechin Pentaacetate

FTIR (cm^−1^): 3024 (stretching vibration of aromatic C-H), 2939 (stretching vibration of aliphatic C-H), 2852 (symmetric stretching vibration of aliphatic C-H), 1764 (stretching vibration of C=O in aryl ester groups), 1739 (stretching vibration of C=O in aliphatic ester group), 1622, 1593, and 1489 (stretching vibrations of aromatic C=C bonds), 1369 (out-of-plane C-H bending vibrations of CH_3_), 1174, 1120, and 1028 (stretching vibrations of C-O bonds), 898, 839, 750, and 665 ((out-of-plane C-H bending vibrations of aromatic rings) Supporting information, Figure S8).

### 3.7. ^1^H NMR of Catechin Pentaacetate

^1^H NMR (500 MHz, CDCl_3_) δ (ppm): 7.25 (dd, *J*_1_ = 8.5, *J*_2_ = 2 Hz, 1 H, aromatic ring), 7.19 (d, *J* = 8.5 Hz, 1 H, aromatic ring), 7.17 (d, *J* = 2.5 Hz, 1 H, aromatic ring), 6.66 (d, *J* = 2.5 Hz, 1 H, aromatic ring), 6.59 (d, *J* = 2.5 Hz, 1 H, aromatic ring), 6.75 (d, *J* = 2.5 Hz, 1 H, aromatic ring), 5.25 (q, *J* = 6.0 Hz, 1 H, aliphatic ring), 5.14 (d, *J* = 6.5 Hz, 1 H, aliphatic ring), 2.86 (dd, *J*_1_ = 16.75, *J*_2_ = 5.5 Hz, 1 H, diastereotopic methylene in aliphatic ring), 2.67 (dd, *J*_1_ = 16.5, *J*_2_ = 6.5 Hz, 1 H, diastereotopic methylene in aliphatic ring), 2.27–2.26 (m, 12 H, four CH_3_ groups), 1.99 (s, 3 H, CH_3_). (Supporting information, Figures S9–S11).

### 3.8. ^13^C NMR of Catechin Pentaacetate

^13^C NMR (125 MHz, CDCl_3_) δ (ppm): 170.13, 168.98, 168.37, 168.07, and 168.06 (carbonyl of ester groups), 154.37, 149.86, 149.42, 142.11, 142.09, 136.14, 124.41, 123.71, 121.78, 110.18, 108.78, and 107.69 (aromatic rings), 77.64, 68.27, and 23.91 (aliphatic ring), 21.10, 20.96, 20.79, 20.65, and 20.63 (methyl groups). (Supporting information, Figure S12).

### 3.9. Particle Sizes of Catechin Pentaacetate

Catechin pentaacetate was dispersed in DI H_2_O using 2 % (*v*/*v*) ethanol as a cosolvent and 0.4 % (*w*/*v*) of tween 80 as a surfactant. The average size of the catechin pentaacetate particles was 509 nm, and the PDI was 0.503.

### 3.10. Gross Physiological Parameters

There were no significant differences in body weight, cecum weight, and cecum-to-body weight ratios across all treatment groups (Table 1).

### 3.11. Hemoglobin Concentration and Glycogen Concentrations of the Pectoral and Hepatic Tissues

There were no significant differences between experimental groups in hemoglobin levels and pectoral and hepatic glycogen content (Table 2).

### 3.12. Cecal Microbiota Analysis

**Figure 1:**
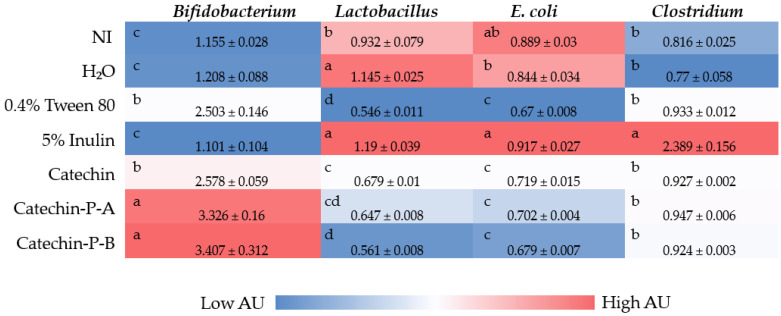
Effects of intra-amniotic injections on cecal genera and species-level bacterial populations (day of hatch). Values are means ± SEM, *n* = 10. ^a–d^ Per bacterial category (in the same column), treatment groups that do not share any letters are significantly different according to a one-way ANOVA with a post hoc Duncan test (*p* < 0.05). NI, No Injection; Catechin-P-A, Catechin pentaacetate; Catechin-P-B, Catechin pentabutanoate.

As demonstrated in Figure 1, *Bifidobacterium* populations were significantly increased (*p* < 0.05) in the catechin pentaacetate (catechin-P-A) and catechin pentabutanoate (catechin-P-B) groups when compared with all other treatment groups. Additionally, there were significant differences between 0.4% Tween 80 and catechin groups compared to the no injection, H_2_O injection, and 5% inulin control groups. It was significantly increased (*p* < 0.05). *Lactobacillus* populations were found in the Catechin-P-A, Catechin-P-B, and 5% inulin groups compared with the catechin, 0.4% Tween 80, H_2_O, and NI groups. The highest *Lactobacillus* density levels were seen in 5% inulin and catechin-P-A administration compared to all other groups (*p* < 0.05). Within *E. coli*, the highest density of bacteria was found within the no injection, H_2_O, and 5% inulin groups compared to 0.4% Tween 80, Catechin, Catechin-P-A, and Catechin-P-B groups (*p* < 0.05). *Clostridium* populations were significantly elevated in the 5% inulin group compared to all the other groups.

### 3.13. Duodenal Gene Expression

#### 3.13.1. Fe-Related Proteins

**Figure 2:**
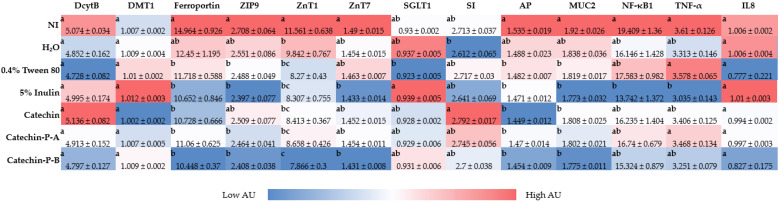
Effect of the intraamniotic administration of experimental solutions on intestinal gene expression. Values are the means ± SEM, *n* = 10. ^a–c^ Per gene (in the same column), treatments groups not indicated by the same letter are significantly different (*p* < 0.05). NI, No Injection; Catechin-P-A, Catechin pentaacetate; Catechin-P-B, Catechin pentabutanoate. DcytB, Duodenal cytochrome B; DMT1, Divalent metal transporter 1; ZIP9, Zinc Transporter 9; ZnT1, Zinc transporter 1; ZnT7, Zinc transporter 7; SGLT-1, Sodium-glucose cotransporter 1; SI, Sucrose isomaltase; AP, amino peptidase; MUC2, Mucin 2; NF-κβ1, Nuclear factor-κβ1; TNF-α, Tumor necrosis factor-alpha; IL8, Interleukin 8; 18S rRNA, 18S Ribosomal subunit.

For the proteins directly responsible for iron uptake, the gene expression for Duodenal cytochrome b (DcytB) and Divalent Metal Transporter 1 was not significantly altered between groups. Ferroportin expression was significantly downregulated (*p* < 0.05, Figure 2) in the 0.4% Tween 80, 5% Inulin, Catechin-P-A, and Catechin-P-B compared to the NI control group.

#### 3.13.2. Zn-Related Proteins

The expression of zinc-related proteins related to cellular uptake, transport, and storage; the gene expression zinc transporter 9 (ZIP9) was significantly down-regulated (*p* < 0.05) with 0.4% Tween 80, 5% Inulin, Catechin, Catechin-P-A, and Catechin-P-B compared with the NI group. Zinc transporter 1 (ZnT1) gene expression was significantly down-regulated (*p* < 0.05, Figure 2) with Catechin-P-B compared to the NI and H_2_O groups. Additionally, zinc transporter 7 (ZnT7) was downregulated considerably (*p* < 0.05, Figure 2) with 5% inulin and catechin-P-B compared to the NI group.

#### 3.13.3. BBM Functionality and Mucin Production Proteins

Sodium-glucose cotransporter 1 (SGLT-1) expression was significantly (*p* < 0.05, Figure 2) upregulated with 5% inulin compared to the 0.4% Tween 80 group. There was a significant upregulation with Catechin compared to the H_2_O injection with Sucrose isomaltase (SI). Amino peptidase (AP) and Mucin 2 (MUC2) were significantly downregulated in the 5% inulin, Catechin, Catechin-P-A and Catechin-P-B groups compared with the NI group.

#### 3.13.4. Pro-Inflammatory Proteins

There was significant downregulation (*p* < 0.05, Figure 2) with 5% inulin compared to the NI groups in nuclear transcription factor-κβ 1(NF-κβ1) expression, and there were no significant differences with the catechin groups compared to the controls. Tumor necrosis factor (TNF-α) had significant downregulation (*p* < 0.05, Figure 2) in 5% inulin when compared to the NI, 0.4% Tween 80, and catechin-P-A groups. There were no significant differences in gene expression of interleukin-8 (IL8) between all the groups.

### 3.14. Duodenal Morphometric Parameters

The catechin and synthetic compounds treatment groups presented elevated (*p* < 0.05) villus surface area and crypt depth versus the controls (NI and H_2_O). Paneth cell diameter and number values were significantly (*p* < 0.05) smaller in catechin, catechin-P-A, and catechin-P-B compared to NI and H_2_O (Table 3).

The villi goblet cell diameter, neutral villi goblet cell, mixed villi goblet cell, and a total number of villi and crypt goblet cells, where catechin and catechin-P-B were significantly smaller than the NI control group. However, the acidic villi goblet cell number of catechin and synthetic catechin compounds were significantly more numerous than the NI and 5% inulin treatment groups. Further, 0.4% tween was significantly higher than all other treatment groups in crypt goblet cell diameter. There was no significant difference between any treatment group in acidic crypt goblet cells per crypt. However, there was a significantly lower number of mixed and neutral goblet cells per crypt of catechin than NI and H_2_O. Furthermore, there was a significantly lower (*p* < 0.05) amount of total goblet cells per crypt in the catechin and synthetic compounds (Catechin-P-A and Catechin-P-B) groups relative to the controls (NI and H_2_O) (Table 4).

## 4. Discussion

Catechin, a flavan-3-ol, is a type of flavonoid and a secondary metabolite of plants that provides antioxidant roles in plants and humans. Although catechin ingestion has been associated with significant beneficial physiological changes related to catechin antioxidant activity [1,4,5,6,7], further understanding of duodenal tissue-level effects associated with catechin exposure is needed. Additionally, the instability of catechin within the digestive tract severely hinders its bioavailability. This study focuses on novel and more stable catechin derivatives, catechin-P-A, and catechin-P-B, synthesized using acetic anhydride and butyric anhydride, respectively. The studied esterification reactions were executed under mild conditions and resulted in acceptable yields, as shown in our previous work [46]. The final purified products were characterized using FTIR, NMR, and LC-MS to confirm their structures, demonstrating the synthesis of catechin pentabutanoate and catechin pentaacetate with high purity. Moreover, we could disperse these compounds in H_2_O for intra-amniotic administration studies.

The effects of synthesized and characterized catechin, catechin-P-A, and catechin-P-B on duodenal BBM development, functionality, and cecal microbial populations were investigated in vivo. These compounds did not significantly affect the body weight, cecum, or to-body weight ratio (Table 1). A potential explanation for this finding is the previous short exposure period in other intra-amniotic administration studies investigating the effects of plant-origin bioactive compounds [44,45,48,52,54].

We studied the effects of the investigated catechin compounds on cecal bacteria populations and found significant differences between genera population densities (Figure 1). Catechin consumption has previously been shown to alter intestinal bacterial populations in vivo, including increasing the populations of beneficial SCFA-producing bacteria [21,22]. We found significant (*p* < 0.05) increases in the *Bifidobacterium* population with catechin derivative exposure (catechin-P-A and catechin-P-B) compared to all other groups. As previously seen by Janiak et al. (2018), catechin (from green tea extract) administration resulted in increased *Bifidobacterium* spp. (increase in colony-forming units/mL) [55]. The increases in *Bifidobacterium* have previously been associated with catechin consumption, where colonic microbiota are hypothesized to metabolize catechin to γ-valerolactones and hippuric acids, which undergo further biotransformation, glucuronidation, sulfation, and methylation within the liver [56,57,58]. Therefore, beneficial bacteria can utilize the catechin and bacterial metabolites as substrates to obtain energy, similar to the effects of growth stimulation observed with inulin and galactooligosaccharides [59,60]. Within the potentially pathogenic bacteria, *E. coli* and *Clostridium* spp., there was a significant (*p* < 0.05) decrease in bacterial density associated with catechin compound administration relative to the controls. Ma et al. (2019) found that certain flavanols (e.g., catechin) can have antimicrobial activity against pathogenic bacteria such as *E. coli* and *Clostridium* [61]. These findings can be attributed to increased SCFA production associated with catechin metabolism by microorganisms within the intestine [62,63,64]. Further, dietary supplementation with catechins and similar compounds could have promoted the expression of serum leptin and induced carbohydrate degradation, which may regulate the presence and abundance of certain bacterial strains [65]. Additionally, the production of beneficial bacteria, *Bifidobacterium*, can improve intestinal barrier function, maintain intestinal homeostasis, and aid in lowering immune response from the host (Paneth cell number and diameter, Table 3).Moreover, we hypothesize that exposure to the SCFA (acetate and butyrate) groups esterified to the catechin derivatives (treatment groups catechin-P-A and catechin-P-B) increased villus surface area and crypt depth (Table 3). Liao et al. (2020) previously investigated the relationship between gut microbiota, SCFAs, and intestinal morphology of growing broilers, where increased acetate exposure was found to increase *Lactobacillus* genera and villus height, which is congruent with our findings [66]. This possibility could explain the significant differences in villi acidic goblet cells between the catechin synthetic compounds and controls in Table 4. It has been shown previously that acetate and butyrate SCFAs mediate intestinal mucosa [67,68,69,70] and epithelial barrier cell proliferation [71,72,73,74].

This maintenance of intestinal homeostasis and immune response can be seen within genotypical responses from key BBM digestive and absorptive proteins, as previously demonstrated through intra-amniotic administration of polyphenols and other plant-origin dietary bioactive compounds [33,36,52,54,75]. In the current study, we investigated catechin and its derivatives on BBM gene expression (Figure 2), and the results demonstrated that catechin, catechin-P-A, and catechin-P-B exposure decreased the expression of ferroportin (transporter of Fe into circulation) and zinc transporters (ZIP9, ZnT7). This decrease in gene expression of ferroportin may be due to the beneficial effects of flavonoids exhibiting antioxidant activity by chelating the redox-active transition metals (e.g., iron) that may act as ROS generators [76,77,78]. Further, catechin and other flavonoids have also been demonstrated to be complex with redox-inactive metals (e.g., zinc), causing alterations in glucidic and lipid metabolisms [76,78,79,80,81,82,83]. Quesada et al. (2011) demonstrated that catechin and other procyanidins displayed an affinity for zinc cations in a solution high enough to dissociate zinc from Zinquin (zinc-specific chelator) both intracellularly (within organelles) and within the cytoplasm [78].

NF-κβ1 is one of the pathways used in the small intestine to respond to microbial dysbiosis because it is a central mediator of immune response found in innate and adaptive immunity [84,85]. When NF-κB1 is stimulated, it translocates within the cell nucleus and is involved in various biological functions, including the release of proinflammatory mediators, such as tumor necrosis factor-alpha (TNF-α) [86,87]. The downregulation of pro-inflammatory proteins (NF-κβ1 and TNF-α) was found with 5% inulin, catechin, catechin-P-A and catechin-P-B exposure compared to the NI control group. This observation agrees with our hypothesis that the intra-amniotic administration of catechin and its synthetic compounds beneficially modulates BBM functionality through positive alterations in cecal microbiota populations, as described above (Figure 1). These findings are similar to the trends in Surh et al. (2001), where catechin and epigallocatechin gallate were shown to inhibit cyclooxygenase-2 and nitric oxide synthase expression, blocking NF-κβ1 activation [88,89]. Paneth cells are key cells within the small intestine that defend the host during innate immunity, and our findings with decreased NF-κβ1 expression are supported by decreases in Paneth cell number and diameter within the crypt (Table 3). Additionally, we can associate the downregulation of these pro-inflammatory proteins with the increased beneficial bacterial populations (Figure 1), which increases overall SCFA production, where SCFA exerts anti-inflammatory effects in the intestinal mucosa (Table 4) because of inhibition by histone deacetylases and activating G-protein coupled receptors as described by Parada Venegas et al. (2019) [90]. Taken together, additional studies are warranted to assess shifts in intestinal functionality, development and microbiota post-hatch and during a long-term feeding trial associated with the consumption of catechin and its more stable derivatives to confirm results within this study. Overall, current results demonstrate a unique approach to evaluate the impact of catechin and its derivatives on BBM functionality markers, the intestinal microbiome and other physiological parameters, as previously demonstrated

Finally, it is important to emphasize that further assessments are necessary to confirm the potential effects of catechin and its derivatives on intestinal morphology and functionality due to potential SCFA production by cecal and/or small intestinal microbiome, as discussed. Overall, current results introduce an innovative approach to evaluating the impact of catechin and its derivatives on BBM functional biomarkers, and intestinal microbial populations, in vivo [46].

## 5. Conclusions

The intra-amniotic administration of physiologically relevant dosages of catechin and its synthetic derivative (catechin pentaacetate and catechin pentabutanoate) on intestinal microbiota populations, brush border membrane morphology, and physiological parameters such as average body weight, cecum weight, hemoglobin concentrations, pectoral and hepatic glycogen concentrations, and duodenal gene expression of newborn chickens are reported. The data suggests that each compound can positively alter duodenal brush border membrane functionality, morphology, and cecal microbial populations (*Bifidobacterium* and *Lactobacillus*). This is one of the first studies to synthesize two synthetically derived catechin esters with increased bioavailability to reveal potential health benefits. Given these findings, these novel catechin esters should be evaluated in additional long-term studies to elucidate the potential health benefits and mechanisms of action.

## Figures and Tables

**Table 1:** Gross physiological parameters measured on the day of hatch ^1^.

Group Name	Average Body Weight (g)	Average Cecum Weight (g)	CW: BW
NI	49.7 ± 1.9 ᵃ	0.39 ± 0.06 ᵃ	0.007 ± 0.001 ᵃ
H_2_O	48.0 ± 2.49 ᵃ	0.43 ± 0.07 ᵃ	0.009 ± 0.002 ᵃ
5 % Inulin	48.3 ± 1.12 ᵃ	0.42 ± 0.11 ᵃ	0.009 ± 0.002 ᵃ
0.4% Tween 80	47.8 ± 1.2 ᵃ	0.40 ± 0.04 ᵃ	0.008 ± 0.001 ᵃ
Catechin	48.6 ± 1.66 ᵃ	0.30 ± 0.03 ᵃ	0.006 ± 0.001 ᵃ
Catechin-P-A	48.1 ± 2.36 ᵃ	0.35 ± 0.06 ᵃ	0.008 ± 0.001 ᵃ
Catechin-P-B	47.5 ± 1.74 ᵃ	0.28 ± 0.05 ᵃ	0.006 ± 0.001 ᵃ

^1^ Values are the means ± SEM, *n* = 10. ^a^ Treatment groups not indicated by the same letter are significantly different (*p* < 0.05). NI, non-injected; CW, cecum weight; BW, body weight; Catechin-P-A, Catechin pentaacetate; Catechin-P-B, Catechin pentabutanoate.

**Table 2:** Hemoglobin, concentrations (g/dL) ^1^.

Group Name	Average Hemoglobin (g/dL)	Average Pectoral Glycogen (mg/mL)	Average Hepatic Glycogen (mg/mL)
NI	9.2 ± 1.2 ᵃ	0.011 ± 0.005 ᵃ	0.3 ± 0.08 ᵃ
H_2_O	9.1 ± 1.3 ᵃ	0.013 ± 0.007 ᵃ	0.56 ± 0.09 ᵃ
5 % Inulin	9.8 ± 1.0 ᵃ	0.008 ± 0.002 ᵃ	0.33 ± 0.13 ᵃ
0.4% Tween 80	12.0 ± 2.1 ᵃ	0.004 ± 0.002 ᵃ	0.35 ± 0.07 ᵃ
Catechin	9.1 ± 1.4 ᵃ	0.005 ± 0.002 ᵃ	0.38 ± 0.07 ᵃ
Catechin-P-A	9.2 ± 1.8 ᵃ	0.006 ± 0.002 ᵃ	0.29 ± 0.05 ᵃ
Catechin-P-B	9.3 ± 0.5 ᵃ	0.007 ± 0.002 ᵃ	0.41 ± 0.11 ᵃ

^1^ Values are the means ± SEM, *n* = 10. ^a^ Treatment group not indicated by the same letter is significantly different (*p* < 0.05). NI = non-injected. Catechin-P-A, Catechin pentaacetate; Catechin-P-B, Catechin pentabutanoate.

**Table 3:** Effect of the intra-amniotic administration of experimental solutions on the duodenal small intestinal villus, crypt depth, and Paneth cells ^1^.

Treatment	Villus Surface Area (µm^2^)	Crypt Depth (µm)	Paneth Cell/Crypt	Paneth Cell Diameter (µm)
NI	35,338.95 ± 814.06 ^d^	22.04 ± 0.66 ^cd^	1.81 ± 0.07 ^b^	1.88 ± 0.10 ^a^
H₂O	36,111.84 ± 735.90 ^d^	21.80 ± 0.54 ^d^	2.02 ± 0.08 ^a^	1.70 ± 0.04 ^b^
0.4% Tween 80	34,700.16 ± 1443.64 ^d^	21.77 ± 1.17 ^d^	1.61 ± 0.05 ^c^	1.46 ± 0.03 ^d^
5% Inulin	38,457.03 ± 1257.71 ^cd^	21.49 ± 0.64 ^d^	2.29 ± 0.09 ^a^	1.64 ± 0.04 ^bc^
Catechin	40,686.07 ± 1337.05 ^bc^	23.58 ± 0.54 ^bcd^	1.65 ± 0.05 ^bc^	1.52 ± 0.03 ^cd^
Catechin-P-A	41,642.87 ± 1189.86 ^abc^	35.04 ± 0.85 ^a^	1.46 ± 0.04 ^c^	1.39 ± 0.03 ^d^
Catechin-P-B	43,200.19 ± 1177.55 ^ab^	25.34 ± 0.98 ^b^	1.51 ± 0.05 ^c^	1.41 ± 0.03 ^d^

^1^ Values are the means ± SEM, *n* = 5. ^a–d^ Treatment groups not indicated by the same letter are significantly different (*p* < 0.05). NI, No Injection; Catechin-P-A, Catechin pentaacetate; Catechin-P-B, Catechin pentabutanoate.

**Table 4:** Effect of the intra-amniotic administration of experimental solutions on the goblet cells ^1^.

Treatment	Villi Goblet Diameter (µm)	Villi Goblet Acid/Villi (#)	Villi Goblet Neutral/Villi (#)	Villi Goblet Mixed/Villi (#)	Villi Goblet Cell (#)	Crypt Goblet Diameter (µm)	Crypt Goblet Acid/Crypt (#)	Crypt Goblet Neutral/Crypt (#)	Crypt Goblet Mixed/Crypt (#)	Crypt Goblet Cell (#)
NI	3.45 ± 0.07 ^d^	31.89 ± 0.89 ^a^	1.85 ± 0.19 ^a^	5.89 ± 0.28 ^a^	39.63 ± 0.93 ^a^	3.24 ± 0.04 ^c^	7.74 ± 0.24 ^a^	1.56 ± 0.24 ^b^	0.86 ± 0.11 ^c^	10.15 ± 0.41 ^ab^
H₂O	3.43 ± 0.06 ^d^	16.27 ± 0.67 ^c^	1.42 ± 0.17 ^b^	5.29 ± 0.72 ^a^	28.94 ± 0.76 ^b^	2.74 ± 0.04 ^e^	7.66 ± 0.22 ^a^	2.62 ± 0.21 ^a^	0.86 ± 0.08 ^c^	11.14 ± 0.35 ^a^
0.4% Tween 80	4.93 ± 0.09 ^a^	34.26 ± 0.95 ^a^	0.04 ± 0.02 ^c^	0.35 ± 0.08 ^d^	30.20 ± 1.24 ^b^	3.95 ± 0.09 ^a^	7.92 ± 0.32 ^a^	0.01 ± 0.01 ^d^	1.32 ± 0.12 ^b^	8.68 ± 0.38 ^cd^
5% Inulin	3.20 ± 0.06 ^e^	23.96 ± 0.68 ^b^	0.08 ± 0.02 ^c^	1.46 ± 0.14 ^c^	25.50 ± 0.75 ^c^	2.18 ± 0.04 ^f^	8.45 ± 0.36 ^a^	0.63 ± 0.11 ^c^	1.70 ± 0.15 ^a^	10.78 ± 0.43 ^a^
Catechin	4.15 ± 0.07 ^b^	32.14 ± 0.68 ^a^	0.01 ± 0.01 ^c^	4.29 ± 0.24 ^b^	29.16 ± 1.19 ^b^	3.72 ± 0.06 ^b^	7.8 ± 0.22 ^a^	0.24 ± 0.04 ^d^	0.38 ± 0.06 ^d^	8.41 ± 0.24 ^d^
Catechin-P-A	3.50 ± 0.05 ^d^	32.36 ± 0.94 ^a^	0.05 ± 0.03 ^c^	3.69 ± 0.28 ^b^	28.89 ± 1.32 ^b^	3.02 ± 0.05 ^d^	7.94 ± 0.26 ^a^	0.07 ± 0.02 ^d^	0.96 ± 0.10 ^c^	8.96 ± 0.30 ^cd^
Catechin-P-B	3.91 ± 0.07 ^c^	33.99 ± 1.02 ^a^	0.10 ± 0.03 ^c^	1.60 ± 0.37 ^c^	24.80 ± 1.41 ^c^	3.27 ± 0.06 ^c^	8.26 ± 0.32 ^a^	0.02 ± 0.01 ^d^	1.25 ± 0.09 ^b^	9.52 ± 0.35 ^bc^

^1^ Values are the means ± SEM, *n* = 5. ^a–f^ Treatment groups not indicated by the same letter are significantly different (*p* < 0.05). NI, No Injection; Catechin-P-A, Catechin pentaacetate; Catechin-P-B, Catechin pentabutanoate. ^#^ Number of cells.

## Supplementary Materials

The following are available online at https://www.mdpi.com/article/10.3390/nu14193924/s1, Figure S1: FTIR of catechin pentabutanoate; Figure S2: ^1^H NMR of catechin pentabutanoate in CDCl_3_; Figure S3: Expanded (7.27–2.64 ppm) ^1^H NMR of catechin pentabutanoate in CDCl_3_; Figure S4: Expanded (2.55–0.84 ppm) ^1^H NMR of catechin pentabutanoate in CDCl_3_; Figure S5: ^13^C NMR of catechin pentabutanoate in CDCl_3_; Figure S6: Expanded ^13^C NMR of catechin pentabutanoate in CDCl_3_; Figure S7: SRM LC-MS of catechin pentabutanoate. (a) SRM LC of catechin pentabutanoate. (b) Mass spectrum of catechin pentabutanoate; Figure S8: FTIR of catechin pentaacetate; Figure S9: ^1^H NMR of catechin pentaacetate in CDCl_3_, Figure S10: Expanded ^1^H NMR of catechin pentaacetate in CDCl_3_; Figure S11: Expanded ^1^H NMR of catechin pentaacetate in CDCl_3_; Figure S12: ^13^C NMR of catechin pentaacetate in CDCl_3_. Table S1: Real-time polymerase chain reaction (RT-PCR) primer sequence.
