# Supplementary material for: Alterations in Intestinal Brush Border Membrane Functionality and Bacterial Populations Following Intra-Amniotic Administration (Gallus gallus) of Catechin and Its Derivatives
Source: Nutrients. 2022 Sep 22;14(19):3924. doi: 10.3390/nu14193924 (PMC9572352; doi:10.3390/nu14193924)
Supplement: Supplementary file 1 [file nutrients-14-03924-s001.zip › nutrients-1917427-supplementary.pdf]

Supplemental Materials

Table S1. Real-time polymerase chain reaction (RT-PCR) primer sequences.

Gene	Forward Primer (5′-3′)	Reverse Primer (5′-3′)	Base Pairs	GI Identifier
		Iron Metabolism		
DcytB	CATGTGCATTCTCTTCCAAAGTC	CTCCTTGGTGACCGCATTAT	103	20380692
DMT1	TTGATTCAGAGCCTCCCATTAG	GCGAGGAGTAGGCTTGTATTT	101	206597489
Ferroportin	CTCAGCAATCACTGGCATCA	ACTGGGCAACTCCAGAAATAAG	98	61098365
		Zinc Metabolism		
ZIP9	CTAAGCAAGAGCAGCAAAGAAG	CATGAACTGTGGCAACGTAAAG	100	237874618
ZnT1	GGTAACAGAGCTGCCTTAACT	GGTAACAGAGCTGCCTTAACT	105	54109718
ZnT7	GGAAGATGTCAGGATGGTTCA	CGAAGGACAAATTGAGGCAAAG	87	56555152
		Inflammatory Response		
NF-κβ1	CACAGCTGGAGGGAAGTAAAT	TTGAGTAAGGAAGTGAGGTTGAG	100	2130627
TNF-α	GACAGCCTATGCCAACAAGTA	TTACAGGAAGGGCAACTCATC	109	53854909
IL8	TCATCCATCCCAAGTTCATTCA	GACACACTTCTCTGCCATCTT	105	395872
		BBM Functionality		
SGLT-1	GCATCCTTACTCTGTGGTACTG	TATCCGCACATCACACATCC	106	8346783
SI	CCAGCAATGCCAGCATATTG	CGGTTTCTCCTTACCACTTCTT	95	2246388
AP	CGTCAGCCAGTTTGACTATGTA	CTCTCAAAGAAGCTGAGGATGG	138	45382360
Muc2	CCTGCTGCAAGGAAGTAGAA	GGAAGATCAGAGTGGTGCATAG	272	423101
18S	GCAAGACGAACTAAAGCGAAAG	TCGGAACTACGACGGTATCT	100	7262899

DcytB, Duodenal cytochrome B; DMT1, Divalent metal transporter 1; ZIP9, Zinc Transporter 9; ZnT1, Zinc transporter 1; ZnT7, Zinc transporter 7; SGLT-1, Sodium-glucose transporter 1; SI, Sucrose isomaltase; AP, amino peptidase; Muc2, Mucin 2; NF-κβ, Nuclear factor- κβ1; TNF-α, Tumor necrosis factor 1; IL8, Interleukin 8; 18S rRNA, 18S Ribosomal subunit.

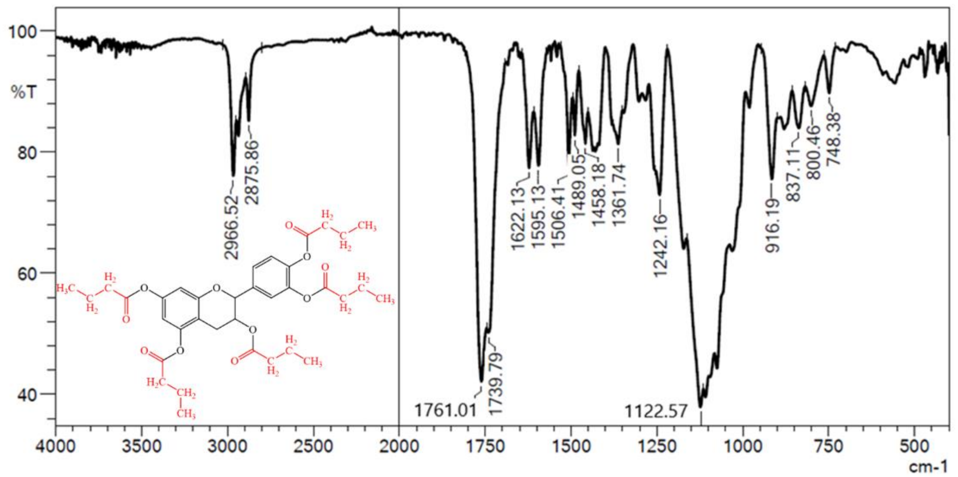

Figure S1. FTIR of Catechin pentabutanoate.

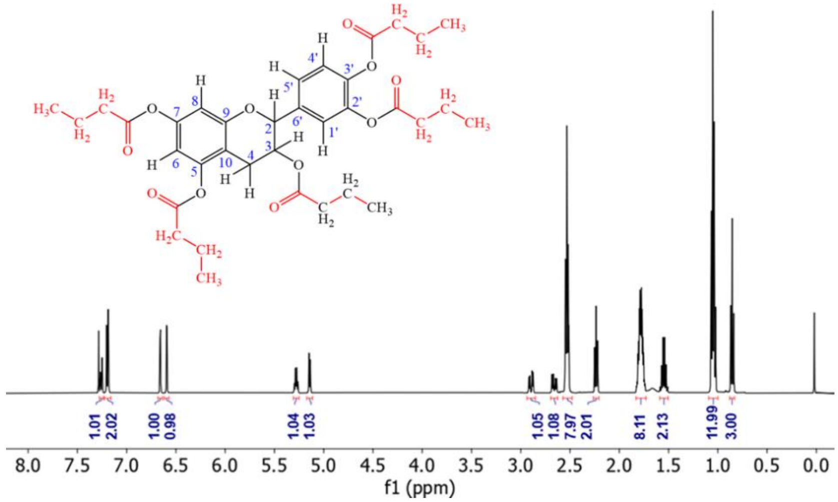

Figure S2. ^{1}H NMR of catechin pentabutanoate in $CDCl_3$.

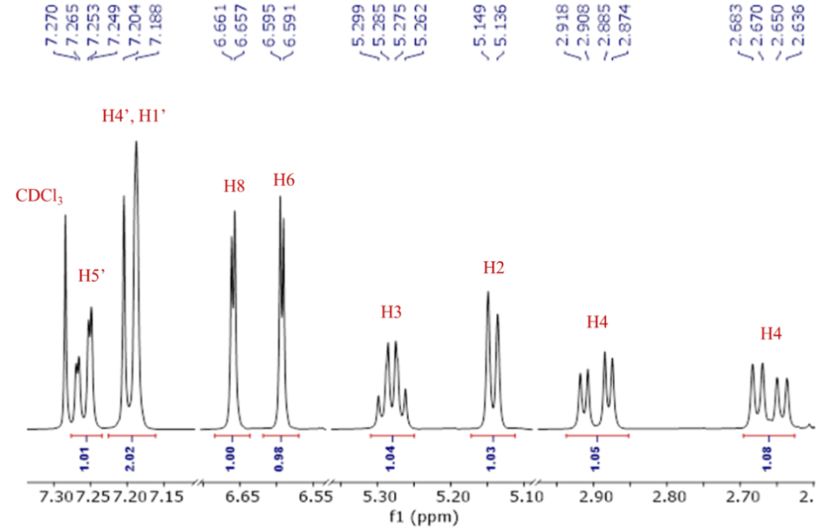

Figure S3. Expanded (7.27-2.64 ppm) ^{1}H NMR of catechin pentabutanoate in $CDCl_3$.

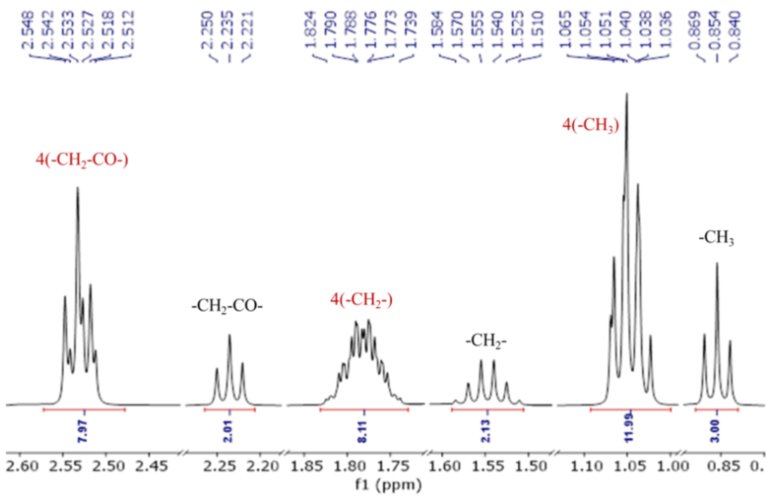

Figure S4. Expanded (2.55-0.84 ppm) ^{1}H NMR of catechin pentabutanoate in $CDCl_3$.

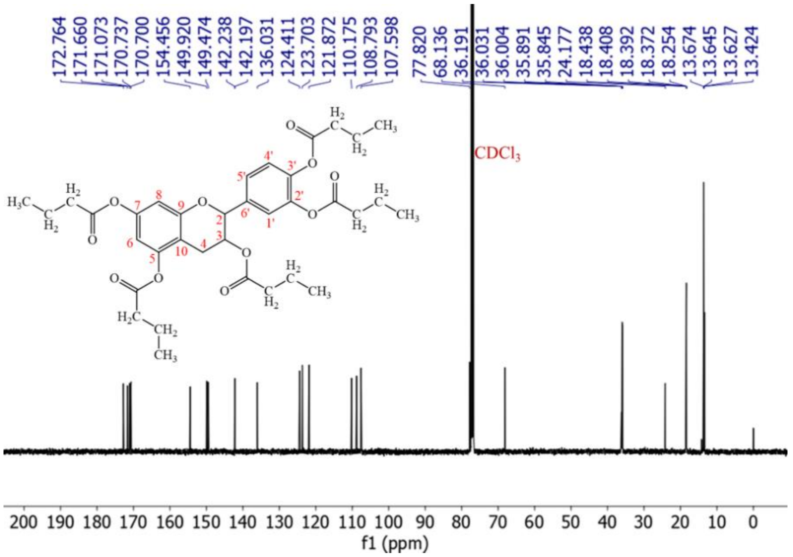

Figure S5. ^{13}C NMR of catechin pentabutanoate in $CDCl_3$.

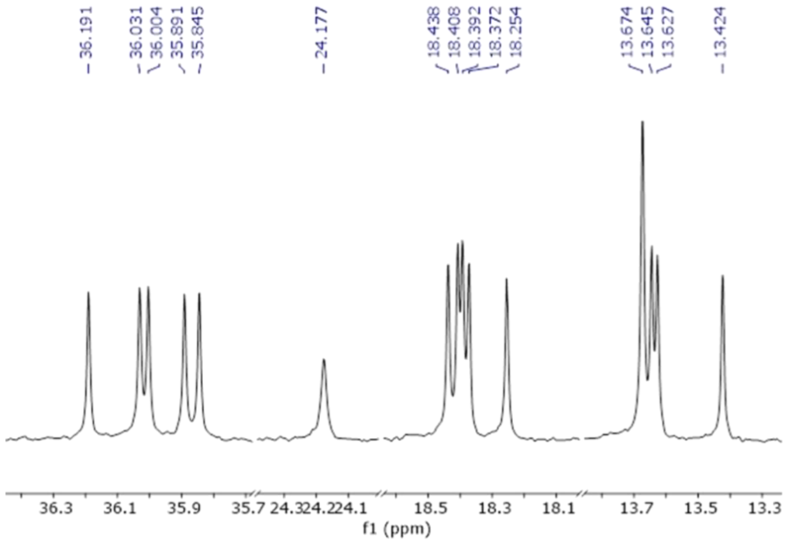

Figure S6. Expanded ^{13}C NMR of catechin pentabutanoate in $CDCl_3$

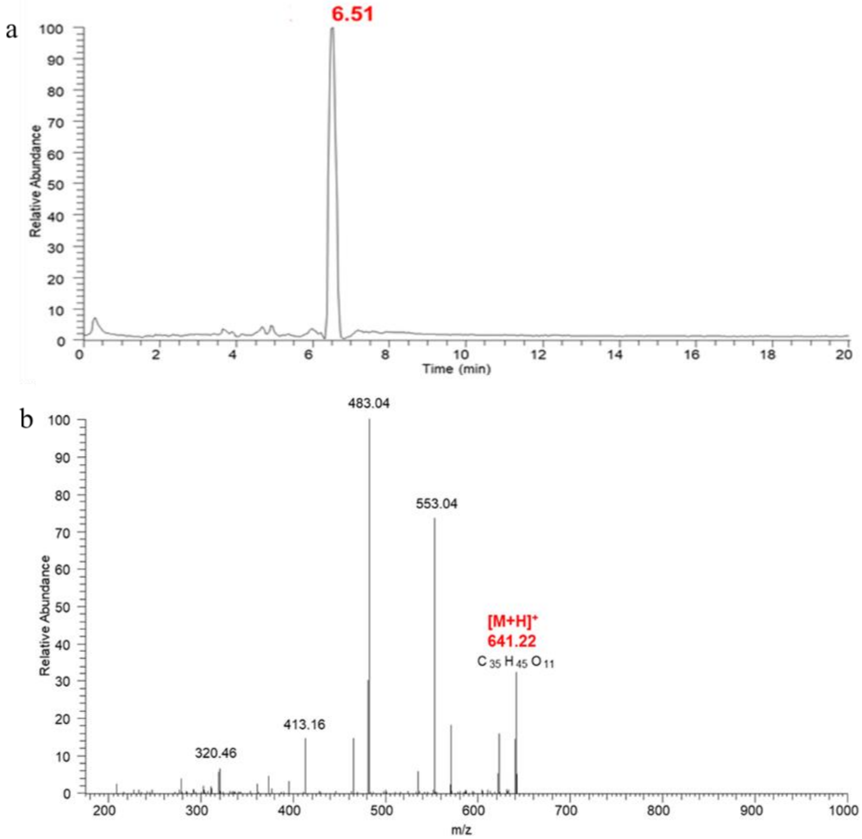

Figure S7. SRM LC-MS of catechin pentabutanoate. a) SRM LC of catechin pentabutanoate. b) Mass spectrum of catechin pentabutanoate.

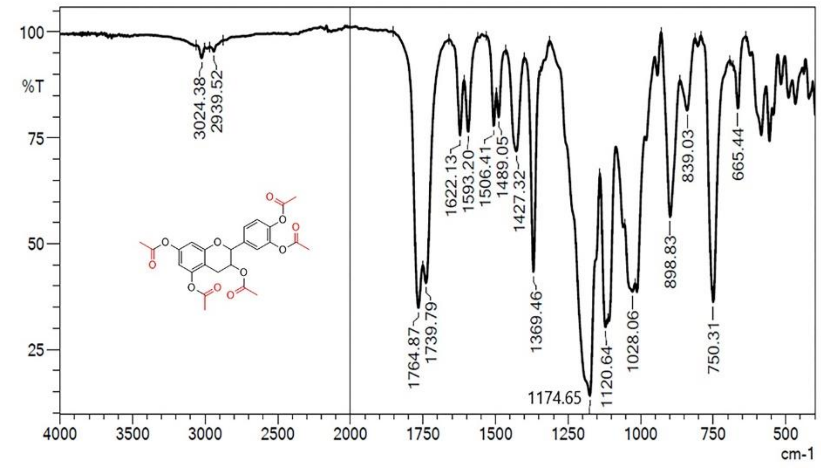

Figure S8. FTIR of Catechin pentaacetate.

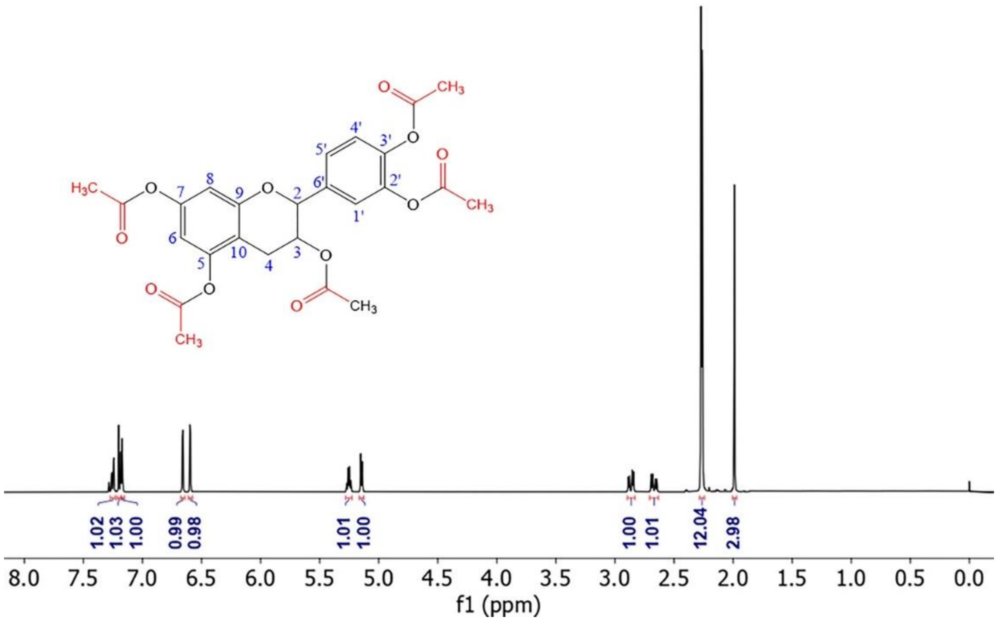

Figure S9. ^{1}H NMR of catechin pentaacetate in $CDCl_3$.

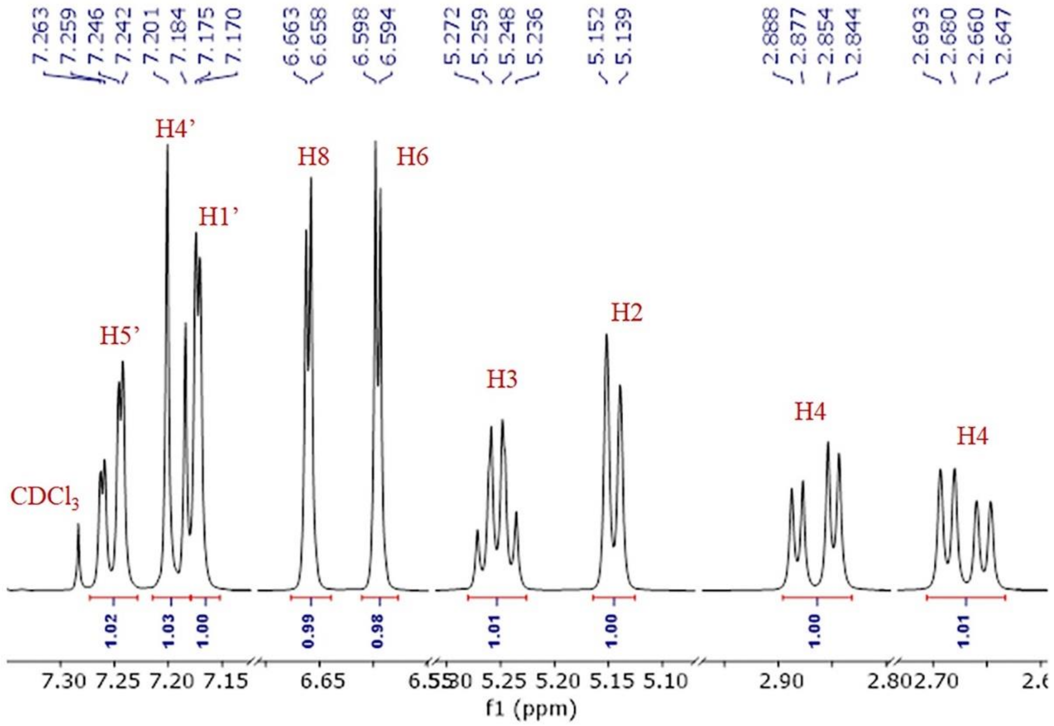

Figure S10. Expanded ^1H NMR of catechin pentaacetate in $CDCl_3$.

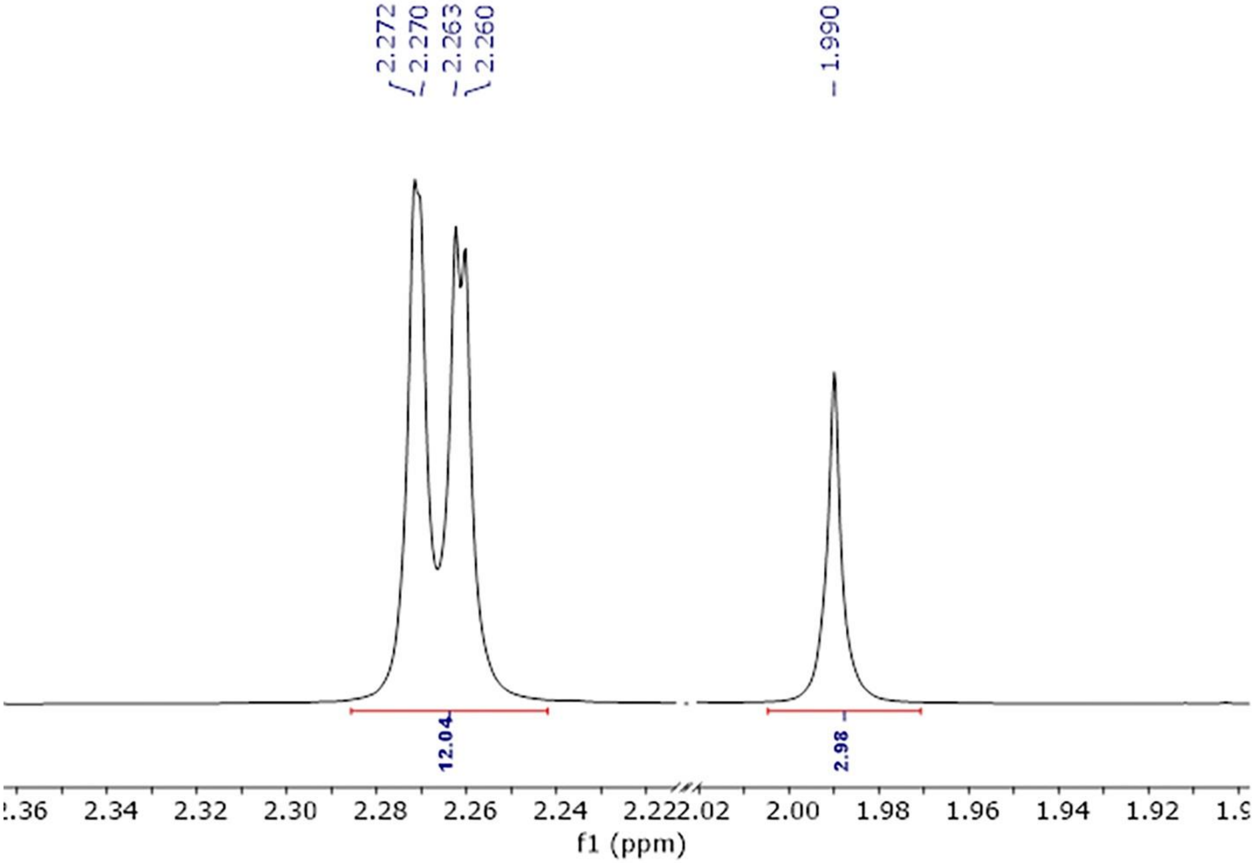

Figure S11. Expanded ^{1}H NMR of catechin pentaacetate in $CDCl_3$.

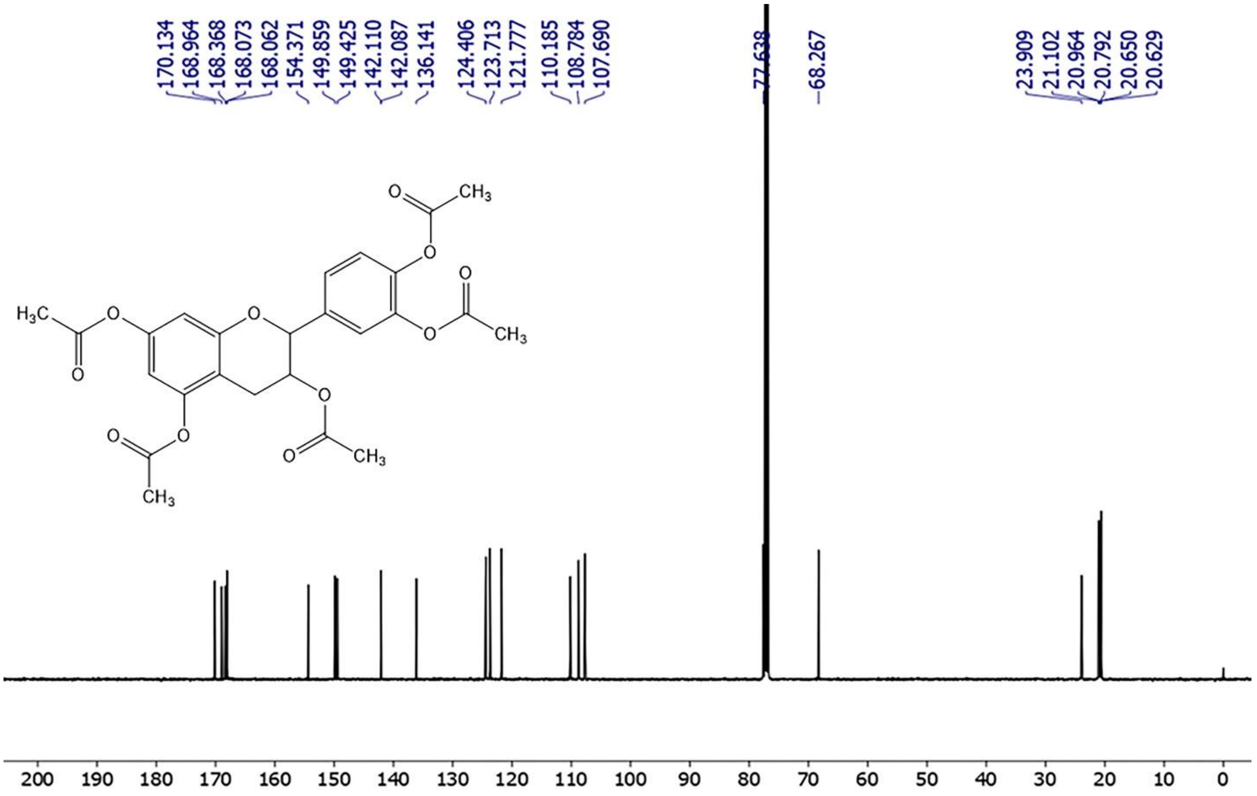

Figure S12. ^{13}C NMR of catechin pentaacetate in $CDCl_3$.
